# Evaluation of Biomechanical Effects of Mandible Arch Types in All-on-4 and All-on-5 Dental Implant Design: A 3D Finite Element Analysis

**DOI:** 10.3390/jfb16040134

**Published:** 2025-04-07

**Authors:** Sema Nur Sevinç Gül, Fahri Murat, Abdullah Tahir Şensoy

**Affiliations:** 1 Department of Periodontology, Faculty of Dentistry, Atatürk University, 25240 Erzurum, Türkiye; sema.sevinc@atauni.edu.tr; 2 Department of Mechanical Engineering, Faculty of Engineering and Architecture, Erzurum Technical University, 25050 Erzurum, Türkiye; fahri.murat@erzurum.edu.tr; 3 Faculty of Mechanical Engineering, Delft University of Technology, Mekelweg 2, 2628 CD Delft, The Netherlands; 4 Department of Biomedical Engineering, Faculty of Engineering and Natural Sciences, Samsun University, 55420 Samsun, Türkiye

**Keywords:** implant biomechanics, mandibular arch morphology, All-on-4, All-on-5, full-arch rehabilitation

## Abstract

This study evaluates the biomechanical effects of different implant configurations in various mandibular arch types using finite element analysis (FEA). Stress distribution and deformation patterns were analyzed under different loading conditions in square, U-shaped, and V-shaped arches. The results indicate that increasing the number of implants generally reduces cortical bone stress, particularly in U and V arches, while implant-level stress tends to increase. Under molar loading, cortical bone stress in the square arch decreased by 16.9% (from 90.61 MPa to 75.27 MPa) with the All-on-5 system, while implant stress in the V arch dropped by 46.26% (from 142.35 MPa to 76.5 MPa). Additionally, the cantilever effect in All-on-4 configurations resulted in higher stress on the prosthesis and implants, particularly in V arches. While the All-on-5 system provided better load distribution, the study highlights the importance of optimizing implant positioning based on mandibular anatomy. Despite limitations such as the use of static forces and standardized arch types, these findings offer valuable insights into the biomechanical performance of full-arch implant rehabilitations, supporting future clinical applications and research.

## 1. Introduction

Dental implants represent a primary treatment modality for individuals experiencing tooth loss, offering significant advantages in both aesthetic and functional outcomes, particularly for fully edentulous patients. However, these patients are highly susceptible to alveolar bone loss due to alveolar ridge resorption. Studies indicate that the resorption process begins immediately after extraction and progresses at its highest rate during the first three to six months, with continued but slower resorption over time [1]. The process remains continuous in the mandible, while it typically stabilizes in the maxilla after approximately ten years [2].

To address this issue, additive bone grafting procedures are frequently employed to enhance both the height and width of the alveolar bone. Nevertheless, such procedures are associated with prolonged treatment durations and substantial financial costs, posing a significant challenge, particularly for elderly patients undergoing dental implant therapy. Despite their high success rates [3], dental implants remain vulnerable to complications such as occlusal trauma and peri-implantitis [4]. Occlusal overload, commonly defined as an excessive occlusal force applied to an implant-supported fixed dental prosthesis, can lead to undue stress on the surrounding bone tissue [5]. Consequently, the biomechanics of dental implants have garnered significant research interest, as scholars aim to optimize implant longevity and clinical outcomes.

The “All-on-4” treatment concept, as introduced by Malo et al. [6], involves the placement of two implants in the anterior segment of the jaw, along with an additional two positioned at an inclination in the posterior segment. This approach utilizes a prosthesis supported by a total of four implants, providing a viable therapeutic option. The use of angulated (or tilted) implants allows for the optimal utilization of the residual jawbone volume, thereby eliminating the need for bone grafting procedures [7]. This technique has been employed to prevent inferior alveolar nerve damage and to minimize the length of prosthetic cantilevers. Furthermore, the inclination of the implants increases the bone-supported portion of the implant, enabling the use of commercially available implants exceeding 10 mm in length and thereby significantly enhancing the bone–implant contact area. The efficacy of this treatment approach has been validated by success rates at three and seven years [8,9,10]. Additionally, Branemark system implants have demonstrated long-term success over a five-year period, as reported by other clinicians who have utilized this system [11].

Mechanical failures are characterized by structural integrity issues, including implant fractures and screw loosening [12]. Biological failures, on the other hand, are pivotal for clinical implications and include conditions such as mucositis and peri-implantitis [13].

Malo et al. further reported that patients who underwent full-arch rehabilitation of the edentulous mandible exhibited a 36.7% incidence of mechanical complications concerning the rehabilitation of both the edentulous maxilla and mandible over a 10- to 18-year follow-up period [14]. A retrospective cohort study with a ten-year follow-up identified mechanical complications in 27.1% of cases and biological complications in 19.8% of patients [15]. While most research has focused on implant-supported prostheses utilizing four, six, or eight implants, relatively few studies have investigated the biomechanical effects of five implants on the mandible [16]. Additionally, numerous finite element analysis studies have been conducted on the All-on-4 system; however, implant applications are frequently evaluated using a standardized mandibular shape.

To the best of our knowledge, this study is the first to examine the biomechanical stress distribution of All-on-4 and All-on-5 implant designs in relation to mandibular arch type. Utilizing finite element analysis, this study investigated stress distribution patterns on the prosthesis, the implant, and the surrounding bone in All-on-4 and All-on-5 implant configurations across three distinct mandibular arch types: square, U, and V arches.

## 2. Materials and Methods

### 2.1. Three-Dimensional Model Generation

The mandibular models were reconstructed using SolidWorks 2024 (SolidWorks Corporation, Concord, MA, USA) based on computed tomography (CT) images from our previous study [17]. Three-dimensional (3D) bone models were developed representing square, triangular, and ovoid mandibular arch designs. The cortical bone layer was designed with an average thickness of 2 mm, while the inner volume was modeled as cancellous bone tissue. Both the cortical and cancellous bone models were exported in stereolithography (STL) format for further processing.

The 4.3 mm × 13 mm Nobel Active System implants (Nobel Biocare AB, Zurich, Switzerland) and multi-unit abutments with 0° (height: 3.5 mm) and 30° (height: 4.5 mm) angulations (Nobel Biocare AB) were scanned using 3Shape A3 (BTech Innovation, Ankara, Turkey) and subsequently exported in STL format. In the All-on-4 implant configuration, two implants with straight (0°) abutments were bilaterally positioned in the canine region of the anterior mandible. In the All-on-5 implant configuration, an additional implant was placed at the midline of the central incisors, while two implants with straight abutments were positioned in the canine region. In both configurations, two posterior implants were placed at an angle of approximately 30° to the occlusal plane and were fitted with 30° angled abutments.

To standardize the design, the distal cantilever length was restricted to 5 mm in all models. Subsequently, all models were accurately positioned with the guidance of experienced clinicians. All solid structures were assumed to be homogeneous, isotropic, and linearly elastic, with material properties such as elastic modulus and Poisson’s ratio specified in Table 1 [18,19,20].

### 2.2. Boundary and Loading Conditions

**Figure 1:**
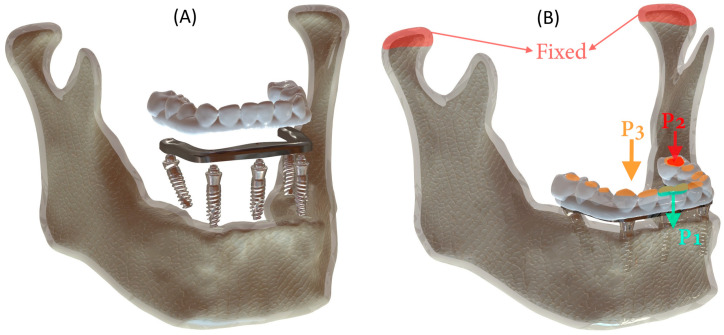
(**A**) 3D model components and (**B**) boundary and loading conditions defined for the mechanical analysis of the model. The directions of the applied P1, P2, and P3 forces are indicated by arrows.

The boundary conditions were defined as the same in all arches. The modeled structures were simulated with tight bonding, and complete osseointegration between the implant and bone was assumed. To simulate anterior (P1) and unilateral posterior (P2) chewing forces, static loads of 100 N were applied [21] (Figure 1). The load magnitude remained constant throughout the simulation. Vertical loading was applied to the central and lateral incisors in the anterior region, while the left second premolar and first molar were subjected to vertical loading in the posterior region. Additionally, a uniformly distributed force (P3) was simultaneously applied to all crowns to facilitate a more insightful comparison between the All-on-4 and All-on-5 designs.

In this study, S4, S5, U4, U5, V4, and V5 are used to denote different arch types and implant configurations, where S, U, and V represent square, U-shaped, and V-shaped arches, respectively, and the numbers 4 and 5 indicate the number of implants (All-on-4 or All-on-5). Additionally, P1, P2, and P3 refer to different loading conditions applied during the simulation. These abbreviations are used throughout the tables and figures to concisely represent each experimental scenario. When all three notations are used together, they specify a unique model. For instance, the notation S4-P1 specifically denotes the All-on-4 configuration within a square arch under the P1 loading condition. On the other hand, combinations of two notations, such as S-P1, represent a set of models that include both implant configurations within a square arch under the P1 loading condition.

### 2.3. Finite Element Analysis

Finite element models were imported into ANSYS 2024R2 software (Ansys Workbench, Canonsburg, PA, USA) for 3D static analysis. First order tetrahedral elements were used to create the mesh structure. The mesh skewness values and the number of elements for each arch shape and implant design are summarized in Table 2, ensuring the reliability and computational accuracy of the finite element models used in this study.

The average mesh size was determined as 1.2 mm for cortical and trabecular bone, 0.3 mm for the implant and bar, and 0.5 mm for the prosthesis. Thus, the average mesh size for all components was 0.7 mm. For mesh sensitivity analysis, the average mesh size was first reduced to 0.5 mm, resulting in over 4,189,032 elements and a skewness value of 0.23 in the S4 model. When the element size was increased to 1 mm, the number of elements decreased to approximately 818,625, and the skewness value exceeded 0.3. Therefore, a mesh size of 0.8 mm, corresponding to approximately 1,244,143 elements and a skewness value of 0.23, was determined to be the optimal parameter. All models were re-meshed again using the optimized mesh parameters in order to ensure consistency.

**Figure 2:**
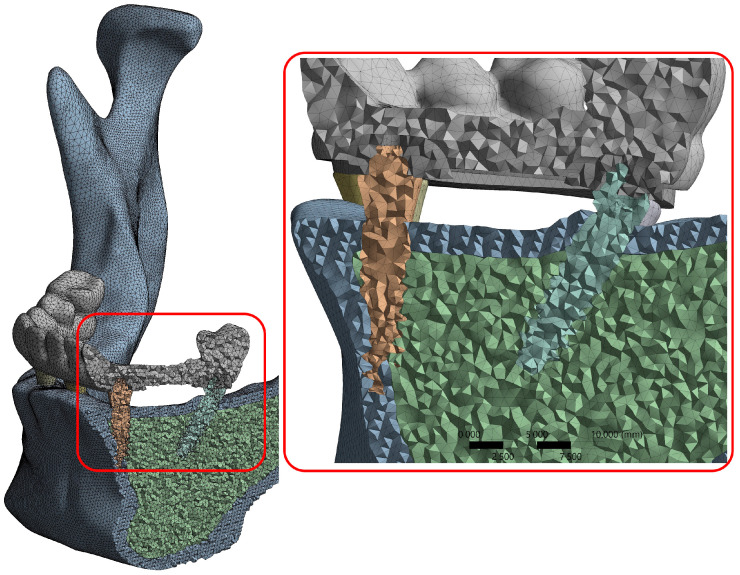
A cross-section from the model to which the mesh process is applied.

A cross-sectional view of the meshed model is illustrated in Figure 2, demonstrating the application of the mesh process and confirming the consistency of element distribution across the structure, which is critical for ensuring the validity of the finite element simulations.

## 3. Results

This study investigates the biomechanical effects of All-on-4 and All-on-5 implant configurations across different mandibular arch types by analyzing stress distribution and deformation under varying loading conditions. The findings provide critical insights into how implant number and arch morphology influence cortical and trabecular bone stress, implant stability, and prosthetic load distribution.

**Figure 3:**
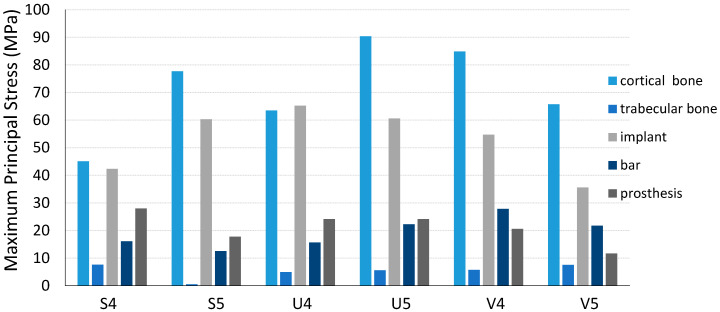
Maximum principal stress of the components of each model under P1 loads.

Figure 3 illustrates the distribution of maximum principal stress (P1) across various components, categorized by arch types and All-on-4 and All-on-5 implant configurations. In cortical bone, the All-on-5 configuration exhibited higher principal stress levels in both square and U arches compared to All-on-4, with the highest stress recorded in the U arch. In trabecular bone, stress values were generally lower than in cortical bone; however, the All-on-4 configuration showed increased stress in the square arch, while the All-on-5 configuration exhibited a slight stress increase in the U and V arches. Regarding the titanium bar, the All-on-4 configuration experienced the highest stress value in the V arch, whereas for the implants, the highest stress was recorded in the All-on-4 configuration in the U arch. Lastly, in the prosthesis, the All-on-4 configuration demonstrated higher stress levels across all arch types compared to the All-on-5 design.

**Figure 4:**
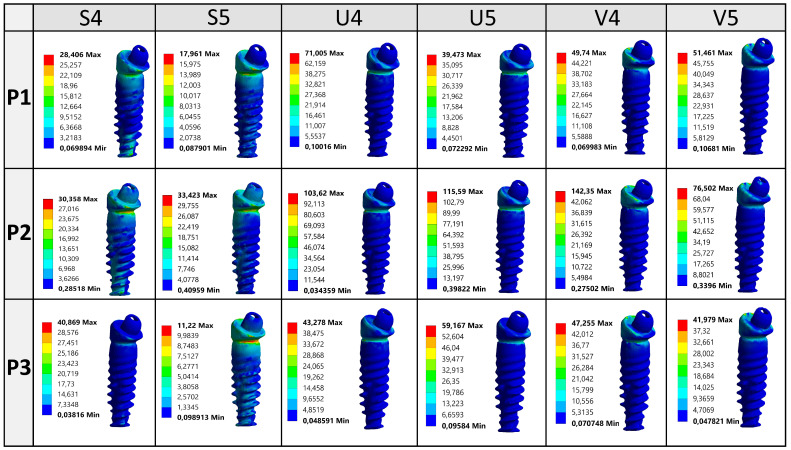
Von Mises stress distribution on the distal implant (36) under P1, P2, and P3 loading.

**Figure 5:**
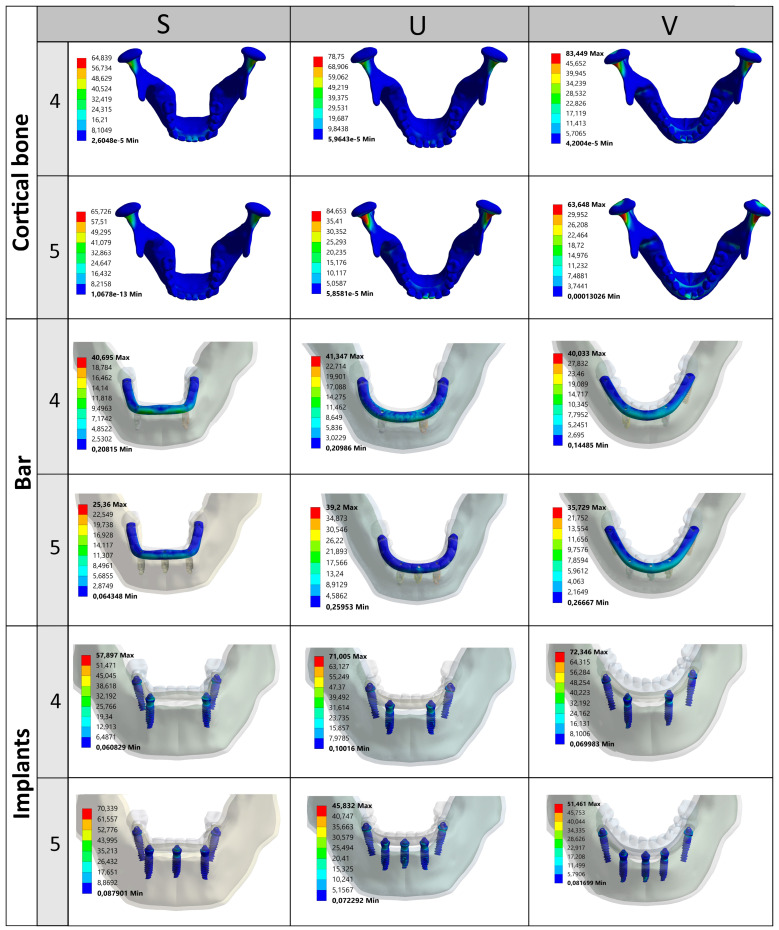
Stress distribution patterns and locations of the maximum von Mises stress for the components under P1 loading.

Table 3, Figure 4 and Figure 5 illustrate the maximum von Mises stress distribution across different components under P1, P2, and P3 loading conditions, categorized by arch types and All-on-4 and All-on-5 configurations. In cortical bone, the All-on-5 design exhibited higher stress levels in the U arch under both loading conditions, whereas in the V arch, stress levels were higher in the All-on-4 configuration at both P1, P2, and P3. Regarding trabecular bone, stress values were generally lower than those in cortical bone. However, in the square arch, the All-on-4 configuration demonstrated relatively higher stress under all loading conditions, while in the V arch, for all loading conditions, von Mises stress was greater in the All-on-5 configuration than in the All-on-4. Moreover, although von Mises stress in the trabecular bone of the U arch was similar in both configurations under P1 and P3 loading, stress levels were notably higher in All-on-5 under P2 loading.

When evaluating the maximum von Mises stress values under all three loading conditions (P1, P2, and P3), distinct and critical differences were observed between the All-on-4 and All-on-5 configurations across all components. In the trabecular bone, the most significant reduction occurred in the S5-P1 model, where stress dropped to 0.66 MPa from 6.01 MPa in S4-P1, indicating an 89% decrease. For the implant component, the highest stress across all scenarios was recorded in the V4-P2 model at 142.35 MPa, whereas the same configuration with All-on-5 (V5-P2) showed a markedly lower value of 76.50 MPa representing a 46% reduction. In contrast, the bar stress peak in the V5-P3 model (44.45 MPa) was significantly higher than the 27.83 MPa seen in V4-P3, suggesting increased lateral transmission in posterior V-shaped arches with All-on-5. Regarding the prosthetic component, the most prominent difference was found under U-P1, where stress in U5-P1 (88.76 MPa) greatly exceeded U4-P1 (54.36 MPa), highlighting a critical drawback of the All-on-5 design in this configuration. Finally, in the cortical bone, the U5-P2 model reached one of the highest stress levels at 96.39 MPa, whereas the V5-P3 model exhibited the lowest value (42.46 MPa), reflecting how both arch shape and load direction critically affect stress distribution. When comparing arch types, square arches generally exhibited more favorable stress values in cortical bone and implant components. For example, under P1 loading, cortical bone stress in S5 was 65.72 MPa, which is 31% lower than U5 (84.65 MPa). Similarly, implant stress in S5-P1 was 28.3% lower than V5-P1 (70.33 MPa vs. 98.27 MPa). In contrast, V-shaped arches (V) tended to show increased bar stress, with the V5-P2 model producing 43% more stress than V4-P2.

Table 4 summarizes the most critical biomechanical differences observed between All-on-4 and All-on-5 implant systems across five anatomical components. Each row highlights a condition (specific load and arch combination) where the stress difference between the two systems was most statistically significant. Notably, trabecular bone exhibited extremely high significance for the model sets of S-P3 and S-P1, indicating that internal bone structure is highly sensitive to implant configuration during posterior loading. The prosthesis and bar components also showed significant stress differences, particularly under S-P1 and S-P3, reinforcing the mechanical importance of arch design in distributing occlusal forces. Meanwhile, cortical bone displayed its highest sensitivity for the sets of the models of U-P2 and S-P2, suggesting that curved arch designs may introduce stress concentration in outer bone regions. Finally, the implant itself showed significant stress variations, especially in S-P2 and U-P1, where the cantilever effect and load direction appear to impact internal stress accumulation. Collectively, this analysis underscores the importance of considering both anatomical component and load direction when planning implant-supported rehabilitations, as implant count and arch shape have measurable effects on biomechanical behavior.

In terms of the titanium bar, the All-on-4 configuration exhibited higher stress levels under P1 loading in most cases, while the All-on-5 design showed increased stress under P2 loading, particularly in the V arch. Regarding implant stress, the highest stress values were observed under P2 loading, with the All-on-4 configuration showing the greatest stress concentration in the V arch, whereas the All-on-5 design exhibited lower stress in the U arch under the same loading condition. Finally, in the prosthesis, stress distribution varied significantly across different arch types. The All-on-4 configuration demonstrated higher stress levels in most arch types, whereas the All-on-5 configuration showed a considerable increase in stress in the U arch under P1 loading.

Figure 4 illustrates the von Mises stress distribution on the distal implant (tooth 36 region) under three different loading conditions (P1, P2, and P3) across various arch forms and implant configurations (four or five implants).

The analysis reveals that stress values tend to increase significantly under P2 loading, especially in the U and V arches, with the highest stress (142.35 MPa) observed in the V4 configuration under P2, suggesting a pronounced cantilever effect. Comparatively, All-on-5 configurations generally exhibit reduced peak stresses, as seen in S5, V5, and U5 across most loading scenarios, confirming the biomechanical advantage of distributing occlusal forces over more implants. Additionally, stress tends to localize around the implant neck, particularly on the buccal side, indicating this region’s mechanical vulnerability. Overall, the data support that both arch form and number of implants significantly influence stress concentration, and optimized implant positioning especially in curved arches can mitigate mechanical overload on distal implants.

Figure 5 presents the stress distribution patterns and highlights the locations of maximum von Mises stress in the cortical bone, bar, and implants under the P1 loading condition.

The evaluation of von Mises stress and deformation results under P1 loading conditions provides critical insights into the relationship between arch types and the number of implants. Under P1 loading, in the square arch, a relatively balanced stress distribution was observed in cortical bone, with stress values increasing slightly by 1.37% from 64.83 MPa to 65.72 MPa in the All-on-5 system. However, in trabecular bone, stress levels significantly decreased by 89%, from 6.01 MPa to 0.66 MPa, indicating improved stress distribution with an increased number of implants. Implant stress also increased by 21.48%, from 57.89 MPa to 70.33 MPa, reflecting a potential concentration of forces within the implants themselves. In the U arch, cortical bone stress was 21.47% higher than in the square arch, reaching 78.75 MPa in the All-on-4 configuration, further increasing by 7.48% in All-on-5. However, implant-level stress significantly decreased by 35.46%, from 71.01 MPa to 45.83 MPa, while stress on the prosthesis increased by 63.29%, from 54.36 MPa to 88.76 MPa. In the V arch, cortical bone stress decreased by 23.73%, from 83.44 MPa to 63.64 MPa, while implant stress dropped by 28.86%, from 72.34 MPa to 51.46 MPa. These findings suggest that increasing implant numbers leads to a reduction in cortical bone stress across all arch types, particularly benefiting load distribution in U and V arches.

Table 5 summarizes the maximum von Mises microstrain values obtained for the implant systems subjected to P1, P2, and P3 loading conditions across three different arch types.

When the results given in Table 5 are examined, it is seen that the analysis of deformation (strain) results under P1, P2, and P3 loading conditions further supports these observations. In the square arch, deformation decreased by 33.46% under P1 loading, from 2156 µε to 1434.7 µε units, and by 6.86% under P2 loading, from 329.26 µε to 306.74 µε units, indicating a relatively stable and balanced distribution of mechanical forces. In the U arch, deformation levels dropped by 49.13%, from 1112.1 µε to 565.48 µε, under P1 loading, whereas a smaller 5.02% reduction was observed under P2 loading, from 1395.9 µε to 1323.5 µε. In the V arch, deformation decreased by 14.92%, from 673.78 µε to 573.92 µε, reflecting a more moderate improvement compared to the square and U arches. Under P3 loading the most notable reduction was observed in the S-P3, where microstrain decreased by approximately 66.6%, from 684.12 µε to 228.65 µε, indicating improved biomechanical stability. These results indicate that increasing the number of implants effectively reduces deformation in cortical and trabecular bone, but the extent of improvement varies across different arch types.

Overall, the square arch demonstrated the most balanced stress and deformation distribution, with reduced cortical and trabecular bone stress when additional implants were placed. The U arch achieved better cortical bone stability, but implant stress increased, especially when more implants were used. In contrast, the V arch exhibited lower cortical bone stress but higher implant stress as the number of implants increased. The findings suggest that while adding more implants generally reduces cortical stress, it may increase stress concentration and deformation at the implant level, particularly in U and V arches, due to greater force transmission through the implants. These insights highlight the importance of optimizing implant placement and prosthetic design to achieve favorable biomechanical outcomes in different mandibular arch configurations.

## 4. Discussion

The results of this research presented notable differences between stress distribution patterns in various mandibular arch configurations when performing the All-on-4 and All-on-5 implant systems. Finite element analysis (FEA) proved that the All-on-4 design would experience high stress values in major structural elements, including the bar, implants, and prosthesis. The increased stress levels considered in All-on-4 systems are due to the cantilever effect, which presents the posterior implants and prosthesis to elevated mechanical stresses. The phenomenon has the potential to result in biomechanical complications, including implant fatigue and prosthesis fractures in the long term [22]. Another critical component of this research is the impact of mandibular arch form on stress distribution. Misch et al. [23] combined three various mandibular arch forms, square, ovoid (U), and angular (V), that all affect anterior and posterior implant distances in the interforaminal area. The current results validate this combination, demonstrating that various arch types considerably changed stress distribution among the implant components.

Recent studies have highlighted the role of arch morphology and anteroposterior (AP) distribution in the biomechanical performance of full-arch implant systems. Narrower arches, such as V-shaped configurations, tend to produce more concentrated stress particularly on distal implants due to their geometric form. In this study, AP distance was kept constant to isolate the effect of arch type, yet V arches still showed higher central stress, indicating that arch shape alone can significantly influence load transfer. These findings align with previous literature showing that arch form affects biomechanical stability [7] and emphasize the importance of adapting implant positioning to mandibular anatomy for optimal stress distribution.

The strategic incorporation of a central incisor implant in configurations such as All-on-5 has been demonstrated to be an effective method of minimizing cantilever effects by reducing leverage arms and mechanical stress on supporting implants [16]. Research findings indicate that constraining cantilever lengths to 1.5 times the AP spread mitigates vertical bone loss, underscoring the significance of implant positioning [24]. Anterior implants have been shown to distribute occlusal forces more evenly, thereby reducing stress concentrations. Conversely, the All-on-4 approach utilizes angled posterior implants to optimize bone utilization and mitigate posterior cantilevering, thereby reducing von Mises stress and the risk of implant failure [25].

Comparatively evaluating All-on-4 and All-on-5 designs, the inclusion of the fifth implant in the All-on-5 configuration yielded a more ideal stress distribution by distributing forces more across the arch. These results are consistent with earlier research recommending extra implant support for situations of excessive occlusal load in order to reduce stress concentration [26,27]. In accordance with the outcomes of the present study, the results of another study conducted by Sun et al. [16] appear to support the biomechanical advantages of the extra implant. The study made a comparison between the efficiencies of All-on-4 and All-on-5 implant designs in an asymmetric mandible, and the All-on-5-o and All-on-5-v designs showed lower stress and strain values compared to All-on-4. On the other hand, the study by Bhering et al. [28] demonstrated that the All-on-6 design provides more favorable stress distribution compared to the All-on-4 concept, suggesting that increasing the number of implants enhances biomechanical stability. Similarly, in a finite element analysis conducted by Aguir et al. [29], the maximum cortical bone stress decreased from 68.6 MPa to 63.9 MPa with the addition of more implants. Takahashi et al. [30] explored how the number of implants affects stress distribution by comparing the traditional All-on-4 design with a six-implant-supported prosthesis. Their findings indicated that using only four parallel implants resulted in approximately 9% more stress compared to the six-implant configuration, primarily due to reduced load-sharing capacity. In a separate ex vivo investigation, Kokat et al. [31] employed strain gauges on cadaveric specimens to measure stress around immediately loaded implants supporting mandibular fixed prostheses. Their results demonstrated a trend of increasing stress with fewer implants under functional loading conditions. In this context, the All-on-5 configuration may serve as an optimized intermediate solution, balancing improved load distribution while minimizing surgical complexity.

The analysis of the effects of the loading conditions provides additional evidence of the influence of arch configuration on stress distribution. The results indicate that P2 loading has a tendency to cause high stress in critical regions, such as implants and cortical bone. This agrees with existing literature demonstrating that occlusal loading at varying locations affects the mechanical load distribution with posterior loading situations resulting in greater distal strain [32]. The results highlight the importance of individualizing implant positioning depending on mandibular anatomy to reduce stress concentrations and improve long-term implant survival. Researchers have also proved that the spatial distance between the loading area and the implant has a profound influence on the von Mises stresses formed within the implants [33]. Loading was applied to the central and lateral incisors in the anterior region and to the left second premolar and first molar teeth in the posterior region. The highest posterior loading stress was noted at the left distal implant for all of the configurations. In the All-on-4 design, the maximum stress concentration was found in the V arch; however, the All-on-5 design showed that as the number of implants increased, the stress concentration around the implants reduced. Furthermore, the lowest strain values were observed in both implant configurations in the square arch at P2.

When assessing screw loosening risks in dental implant systems, different arch types such as square, U, and V configurations provide unique insights. All-on-4 and All-on-5 systems both yield microstrain values under the critical threshold of 4000 µε, implying a general safety against screw loosening under standard loading conditions [34]. However, variations exist across arch types; for instance, U types demonstrate lower microstrain under the P1 loading condition for the All-on-5 design, potentially indicating a reduced risk for screw loosening when coupled with adequate bone density. In contrast, for the same loading condition, V arch configurations reveal higher strain levels, increasing the potential for mechanical failure; this could suggest a greater need for careful monitoring and possibly a preventative approach in patient management. Moreover, implant stability is significantly influenced by various factors, including the chosen materials and implant geometry. As such, models that exhibit better stress distribution in the context of low microstrain highlight superior performance and longevity [17]. Therefore, patient-specific parameters, such as bone quality and morphology, coupled with arch configurations and material properties, should be meticulously considered when assessing the risk of screw loosening across different implant systems as well as various mandibular arch types.

In this study, the All-on-4 design was adjusted such that the anterior teeth were relocated from the lateral positions to that of the canines. Previous studies have shown that positioning the anterior implants in the canine site can enhance stress distribution favorably. In one study, the anterior implant position was altered from the central incisor to the canine in the All-on-4 configuration in the mandible, which led to a decrease in the maximum stress on the peri-implant bone from 68.6 MPa to 63.9 MPa [35]. Furthermore, it has been confirmed that placing the anterior implants in the canine zone in the corner of the arch can lead to a reduction in stress. This placement has the effect of improving the distribution of forces by shortening the distance between the anterior implants and the applied load in comparison to the conventional All-on-4 design [36]. These results are in line with our findings, where a transition from an All-on-4 to an All-on-5 configuration led to a decrease in stress from 90.61 MPa to 75.27 MPa (a 16.9% reduction). Although absolute stress values may differ due to variations in modeling parameters, arch morphology, and loading conditions, both studies clearly demonstrate the biomechanical advantage of increasing implant number in terms of stress distribution. This suggests that, especially under posterior loading conditions, All-on-5 systems may help reduce stress accumulation not only in cortical bone but also in implant–prosthesis components. Moreover, the trabecular bone stress values observed in this study were well below the commonly accepted physiological tolerance threshold of 10 MPa [37]. For example, the All-on-5 square arch configuration yielded a trabecular stress of only 1.37 MPa, indicating minimal risk of overload and supporting long-term bone preservation.

In evaluating the outcomes of our study in relation to defined bone failure limits, it should be noted that cortical bone stress above 140 MPa poses a risk of resorption [38]. For the implant–bone complex to function optimally and maintain physiological balance, stress must remain within a certain range, with intraosseous strains typically falling between 100 and 1500 με. If strains rise to the 1500–3000 με range, the bone experiences slight overload, leading to remodeling as a natural response to repair and adapt to the load [16]. In this study, the All-on-4 U arch configuration was found to generate a maximum cortical bone stress of 96.39 MPa under P2 loading, indicating that it remains well within safe limits. On the other hand, the All-on-4 V arch was observed to reach 142.35 MPa, approaching the critical threshold and suggesting a greater risk of resorption. Meanwhile, the All-on-4 square arch configuration exhibited a maximum cortical bone stress of 90.61 MPa, reinforcing its safety in terms of bone health. These findings align with previous research, where the importance of maintaining stress levels below critical thresholds has been emphasized to prevent bone resorption and ensure the long-term stability of implant-supported prostheses. Although the All-on-4 technique is still a valid treatment option, its biomechanical restrictions require cautious evaluation, especially in individuals with unfavorable configurations of the mandibular arch.

The analysis of microstrain values in Table 5 reveals that most implant systems across different arch types and loading conditions remain within the physiologically acceptable range of 100–1500 µε, promoting favorable bone remodeling. However, certain configurations demonstrate elevated strain levels that warrant clinical consideration. Notably, the S4-P1 model exhibited the highest microstrain value (2156.00 µε, implant 43), significantly exceeding the optimal threshold. Similarly, the V5-P1 model showed a strain value of 1540.60 µε (Implant 36), which falls within the mild overload range (1500–3000 µε). Although S5-P1 (1434.70 µε) remained below the 1500 µε threshold, its proximity to the upper limit suggests the need for cautious evaluation in clinical applications. These elevated strain levels may induce adaptive bone remodeling, but prolonged exposure could lead to undesirable bone resorption or implant instability. Consequently, clinicians should prioritize close monitoring of these specific cases and consider modifications in implant design or loading protocols to ensure long-term biomechanical compatibility. Strains for all other models are within the optimal range, indicating their suitability for clinical use under the tested conditions.

The transfer of load and its impact on stress distribution between dental implants and the surrounding bone have been key areas of focus in FEA studies. Moreover, FEA has proven to be an effective tool for assessing and enhancing the biomechanical performance of multi-implant treatments, such as the All-on-4 and All-on-5 approaches [39]. By simulating various parameters within a design model, FEA enables the exploration of their potential significance in real-world clinical applications. In the present study, cone-beam computed tomography data for the mandible model was obtained from a suitable patient and the model was redesigned with software according to square, U, and V arch types. Furthermore, implants and abutments were scanned using a 3D scanner and transferred to the computer environment in STL format. FEA was applied to investigate the stress distribution in bone and implants in All-on-4 and All-on-5 systems according to square, U, and V arch types of the mandible.

In the digital models developed for this study, the number of elements ranged between 1,244,143 and 2,105,435, while the mesh skewness varied between 0.2 and 0.23. These values were considered sufficient to maximize the accuracy of mechanical analysis when compared to similar studies [40,41].

The following recommendations for clinical practice are supported by the findings of this study: In V-shaped arches, the increased cantilever effect and higher stress concentrations observed in All-on-4 configurations highlight the importance of considering an All-on-5 approach whenever feasible. This configuration may provide improved biomechanical stability by mitigating excessive prosthetic stress. Additionally, the distal placement of posterior implants, within the limits of anatomical structures, can further optimize load distribution and enhance long-term treatment outcomes. For U-shaped arches, All-on-5 offers a biomechanical advantage by reducing cortical bone stress. However, when anatomical limitations or financial constraints preclude its use, All-on-4 remains a viable option, provided that the anterior–posterior spread is maximized to improve load distribution. In square-shaped arches, both All-on-4 and All-on-5 demonstrate favorable biomechanical performance, suggesting that either configuration may be appropriate. Nonetheless, All-on-5 appears to provide superior load distribution, making it a preferable choice in cases involving bruxism or high occlusal forces. These findings offer clinicians valuable insights for optimizing full-arch implant rehabilitation based on mandibular morphology. Tailoring treatment strategies to arch shape may enhance prosthetic longevity, improve load management, and contribute to more predictable clinical outcomes.

The limitations of the study can be categorized in several ways. Firstly, restricting the mandible to three different standard arch types is a major limitation, as it prevents the determination of an ideal arch type due to the unique characteristics of each arch and the challenge of defining a specific form. Additionally, the study was designed as a clinical practice guideline without performing detailed calculations related to arch types. However, the patient’s mandibular structure can be evaluated by the relevant clinician to determine which of the three selected arch types it most closely resembles, allowing for an approximate optimal treatment plan. Secondly, the use of static forces instead of dynamic forces represents another limitation. Furthermore, the model used in this study was assumed to be linearly elastic, homogeneous and isotropic, which does not fully reflect the complex biomechanical properties of the mandible. Despite these limitations, future longitudinal studies be conducted in order to further investigate the biomechanical behavior of All-on-4 and All-on-5 implant designs in different arch types. Such studies would be improved by the incorporation of additional follow-up assessments at various time points (e.g., six months, one year, and beyond). Furthermore, conducting advanced finite element analyses and in vivo studies to analyze the impact of factors such as bone density, implant angulation, and prosthetic material could provide deeper insights into the long-term stability and load distribution of these designs. Combining numerical simulations with clinical follow-ups would further enhance our understanding of the clinical relevance of these findings.

## 5. Conclusions

This study demonstrates that stress distribution in All-on-4 and All-on-5 implant configurations is significantly influenced by mandibular arch morphology and loading conditions. Increasing the number of implants generally reduced cortical bone stress while leading to higher implant-level stress, particularly in U and V arches. These findings suggest that implant configurations should be carefully selected based on the patient’s arch type to optimize load distribution and minimize biomechanical complications.

The All-on-4 design exhibited higher stress levels in key structural components, particularly in the prosthesis and implants, due to the cantilever effect. In contrast, the All-on-5 design distributed occlusal forces more evenly, reducing cortical bone stress while slightly increasing implant stress. These results emphasize the importance of individualized treatment planning, particularly for patients with angular arch morphology or increased occlusal loading, where additional implant support may be beneficial.

While finite element analysis provides valuable insights into the biomechanical behavior of implant-supported prostheses, further long-term clinical studies are necessary to validate these findings. Future research should focus on assessing implant longevity, prosthetic complications, and patient-reported outcomes to refine treatment protocols and improve long-term success in full-arch implant rehabilitations.

## Figures and Tables

**Table 1:** Material properties in the finite element model.

Component Material [Ref]	Elastic Modulus (GPa)	Poisson’s Ratio
Cortical bone [18]	13.70	0.30
Trabecular bone [18]	1.37	0.30
Titanium (implants) [18]	115	0.35
Ti-6Al-4V alloy (bar) [19]	110	0.33
Feldspathic porcelain [20]	82.8	0.35

**Table 2:** Table showing mesh skewness and number of elements according to arch shapes and implant designs.

Arch Type	S		U		V	
Implant System	All-on-4	All-on-5	All-on-4	All-on-5	All-on-4	All-on-5
Skewness Ratio	0.23	0.23	0.21	0.21	0.23	0.2
Number of nodes	1,881,325	1,648,657	3,098,999	2,726,375	2,066,018	1,967,570
Number of elements	1,244,143	1,128,018	2,105,435	1,831,789	1,396,877	1,340,489

**Table 3:** The table of maximum von Mises stress distributions for components.

Component	Implant System	S			U			V		
		P1	P2	P3	P1	P2	P3	P1	P2	P3
Cortical bone	All-on-4	64.83	90.61	92.61	78.75	90.62	70.49	83.44	54.86	69.03
	All-on-5	65.72	75.27	95.49	84.65	96.39	95.93	63.64	42.46	52.75
Trabecular bone	All-on-4	6.01	3.48	6.25	4.25	5.68	3.69	4.88	3.06	3.99
	All-on-5	0.66	1.37	0.58	4.69	7.28	4.14	6.44	5.61	6.92
Bar	All-on-4	40.69	18.78	22.41	41.34	24.26	26.41	40.03	31.09	27.83
	All-on-5	25.36	25.75	10.31	39.21	34.87	25.76	35.72	44.45	22.63
Implant	All-on-4	57.89	30.35	40.86	71.01	103.6	43.27	72.34	142.35	47.25
	All-on-5	70.33	33.42	25.44	45.83	115.5	59.16	51.46	76.50	41.97
Prothesis	All-on-4	52.29	13.68	14.22	54.36	10.57	8.72	27.92	17.12	15.93
	All-on-5	30.75	15.52	10.08	88.76	16.39	5.71	16.59	13.59	10.77

All units are in MPa.

**Table 4:** Top two statistically significant results per component (All-on-4 vs. All-on-5 comparison).

Component	Model Set Compared	t-Statistic	*p*-Value
Trabecular bone	S-P3	66.70	2.10 × 10^−32^
Trabecular bone	S-P1	36.79	2.97 × 10^−25^
Prosthesis	V-P1	28.18	4.29 × 10^−22^
Prosthesis	S-P1	23.26	7.49 × 10^−20^
Bar	S-P1	22.83	1.22 × 10^−19^
Bar	S-P3	20.74	1.36 × 10^−18^
Cortical bone	U-P2	−7.29	3.61 × 10^−7^
Cortical bone	S-P2	−6.30	1.69 × 10^−6^
Implant	S-P2	5.75	5.61 × 10^−6^
Implant	U-P1	−5.26	1.32 × 10^−5^

Note: Model sets were compared in terms of maximum von Mises stress values.

**Table 5:** Maximum von Mises microstrain values obtained for the implant systems.

Arch Type	S			U			V		
Loading Condition [Microstrain]	P1 [µε]	P2 [µε]	P3 [µε]	P1 [µε]	P2 [µε]	P3 [µε]	P1 [µε]	P2 [µε]	P3 [µε]
All-on-4	2156.00	329.26	684.12	1112.10	1395.90	560.17	673.78	573.92	492.91
(Implant No.)	(43)	(36)	(36)	(43)	(36)	(36)	(43)	(36)	(36)
All-on-5	1434.70	306.74	228.65	565.48	1323.50	685.08	1540.60	917.27	461.45
(Implant No.)	(0)	(36)	(36)	(46)	(36)	(36)	(36)	(36)	(36)
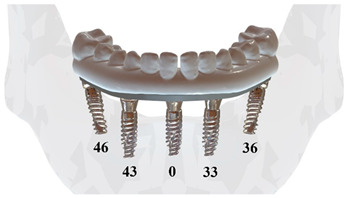									

## Data Availability

The raw data supporting the conclusions of this article will be made available by the authors on request.

## References

[1] Araújo M.G., Lindhe J. (2005). Dimensional ridge alterations following tooth extraction. An experimental study in the dog. J. Clin. Periodontol..

[2] Kovaëiê I., Elebiê A., Kovaëiê F., Knezoviê-Zlatariê D., Bauëiê M., Mehuliê K. (2024). Influence of Night-Time of Denture Wearing on the Rate of Alveolar Ridge Resorption in Complete Denture Wearers. A One-Year Study. Acta Stomatol. Croat..

[3] Kaur M., Abou-Arraj R.V., Lin C.P., Geisinger M.L., Geurs N.C. (2021). A 5-Year Retrospective Analysis of Biologic and Prosthetic Complications Associated with Single-Tooth Endosseous Dental Implants: Practical Applications. Clin. Adv. Periodontics.

[4] Di Fiore A., Montagner M., Sivolella S., Stellini E., Yilmaz B., Brunello G. (2022). Peri-Implant Bone Loss and Overload: A Systematic Review Focusing on Occlusal Analysis through Digital and Analogic Methods. J. Clin. Med..

[5] Graves C.V., Harrel S.K., Rossmann J.A., Kerns D., Gonzalez J.A., Kontogiorgos E.D., Al-Hashimi I., Abraham C. (2016). The Role of Occlusion in the Dental Implant and Peri-implant Condition: A Review. Open Dent. J..

[6] Maló P., Rangert B., Nobre M. (2003). ‘All-on-Four’ Immediate-Function Concept with Brånemark System^®^ Implants for Completely Edentulous Mandibles: A Retrospective Clinical Study. Clin. Implant Dent. Relat. Res..

[7] Del Fabbro M., Bellini C.M., Romeo D., Francetti L. (2012). Tilted implants for the rehabilitation of edentulous jaws: A systematic review. Clin. Implant Dent. Relat. Res..

[8] Maló P., De Araújo Nobre M., Lopes A., Francischone C., Rigolizzo M. (2012). ‘All-on-4’ Immediate-Function Concept for Completely Edentulous Maxillae: A Clinical Report on the Medium (3 Years) and Long-Term (5 Years) Outcomes. Clin. Implant Dent. Relat. Res..

[9] Maló P., De M., Nobre A., Lopes A., Ferro A., Gravito I. (2014). All-on-4® Treatment Concept for the Rehabilitation of the Completely Edentulous Mandible: A 7-Year Clinical and 5-Year Radiographic Retrospective Case Series with Risk Assessment for Implant Failure and Marginal Bone Level. Clin. Implant Dent. Relat. Res..

[10] Baggi L., Pastore S., Di Girolamo M., Vairo G. (2013). Implant-bone load transfer mechanisms in complete-arch prostheses supported by four implants: A three-dimensional finite element approach. J. Prosthet. Dent..

[11] Balshi T.J., Wolfinger G.J., Slauch R.W., Balshi S.F. (2014). A Retrospective Analysis of 800 Brånemark System Implants Following the All-on-Four^TM^ Protocol. J. Prosthodont..

[12] Zhou L., Su Y., Wang J., Wang X., Liu Q., Wang J. (2022). Effect of exposure rates with customized versus conventional titanium mesh on guided bone regeneration: Systematic review and meta-analysis. J. Oral Implantol..

[13] Chrcanovic B.R., Kisch J., Larsson C. (2020). Analysis of technical complications and risk factors for failure of combined tooth-implant-supported fixed dental prostheses. Clin. Implant Dent. Relat. Res..

[14] Maló P., de Araújo Nobre M., Lopes A., Ferro A., Botto J. (2019). The All-on-4 treatment concept for the rehabilitation of the completely edentulous mandible: A longitudinal study with 10 to 18 years of follow-up. Clin. Implant Dent. Relat. Res..

[15] Grandi T., Signorini L. (2022). Rehabilitation of the Completely Edentulous Mandible by All-on-Four Treatment Concept: A Retrospective Cohort Study with Up to 10 Years Follow-Up. Medicina.

[16] Sun X., Cheng K., Liu Y., Ke S., Zhang W., Wang L., Yang F. (2023). Biomechanical comparison of all-on-4 and all-on-5 implant-supported prostheses with alteration of anterior-posterior spread: A three-dimensional finite element analysis. Front. Bioeng. Biotechnol..

[17] Sensoy A.T., Çolak M., Kaymaz I., Findik F. (2021). An application of finite element method in material selection for dental implant crowns. Biomed. Tech..

[18] Türker N., Büyükkaplan U.S., Sadowsky S.J., Özarslan M.M. (2019). Finite Element Stress Analysis of Applied Forces to Implants and Supporting Tissues Using the ‘All-on-Four’ Concept with Different Occlusal Schemes. J. Prosthodont..

[19] Kilic E., Doganay O. (2020). Evaluation of Stress in Tilted Implant Concept with Variable Diameters in the Atrophic Mandible: Three-Dimensional Finite Element Analysis. J. Oral Implantol..

[20] Liu T., Mu Z., Yu T., Wang C., Huang Y. (2019). Biomechanical comparison of implant inclinations and load times with the all-on-4 treatment concept: A three-dimensional finite element analysis. Comput. Methods Biomech. Biomed. Eng..

[21] Mericske-Stern R., Zarb G.A. (1996). In vivo measurements of some functional aspects with mandibular fixed prostheses supported by implants. Clin. Oral Implant Res..

[22] Berzaghi A., Testori T., Scaini R., Bortolini S. (2025). Occlusion and Biomechanical Risk Factors in Implant-Supported Full-Arch Fixed Dental Prostheses—Narrative Review. J. Pers. Med..

[23] Resnik R. (2021). Misch’s Contemporary Implant Dentistry.

[24] Bhering C.L.B., Mesquita M.F., Kemmoku D.T., Noritomi P.Y., Consani R.L.X., Barão V.A.R. (2016). Comparison between all-on-four and all-on-six treatment concepts and framework material on stress distribution in atrophic maxilla: A prototyping guided 3D-FEA study. Mater. Sci. Eng. C Mater. Biol. Appl..

[25] Aguir H., Mabrouk Y., Chamekh R., Saadellaoui I. (2023). Influence of implants number on the biomechanical behavior of implant-supported complete prosthesis: A 3D finite element analysis. Heliyon.

[26] Takahashi T., Shimamura I., Sakurai K. (2010). Influence of number and inclination angle of implants on stress distribution in mandibular cortical bone with All-on-4 Concept. J. Prosthodont. Res..

[27] Kökat A.M., Cömert A., Tekdemir I., Akkocaoǧlu M., Akça K., Çehreli M.C. (2009). Human ex vivo bone tissue strains around immediately-loaded implants supporting mandibular fixed prostheses. Implant Dent..

[28] Kammoun Y., El sharkawy A., Khamis M., Fahmy R. (2022). Radiographic Assessment of Bone Level Changes Around Four Implants Supporting Immediately Loaded Mandibular Screw-retained Prosthesis. Alex. Dent. J..

[29] Ozan O., Kurtulmus-Yilmaz S. (2018). Biomechanical Comparison of Different Implant Inclinations and Cantilever Lengths in All-on-4 Treatment Concept by Three-Dimensional Finite Element Analysis. Int. J. Oral Maxillofac. Implants.

[30] Rangert B., Jemt T., Jörneus L. (1989). Forces and moments on Branemark implants. Int. J. Oral Maxillofac. Implants.

[31] Krekmanov L., Kahn M., Rangert B., Lindström H. (2000). Tilting of posterior mandibular and maxillary implants for improved prosthesis support. Int. J. Oral Maxillofac. Implants.

[32] Valente F., Marrocco A., Falcinelli C. (2025). Impact of physiological and non-physiological loading scenarios and crown material on periimplant bone stress distribution: A 3D finite element study. J. Mech. Behav. Biomed. Mater..

[33] Almeida E.O., Rocha E.P., Freitas J.A.C., Anchieta R.B., Poveda R., Gupta N., Coelho P.G. (2015). Tilted and short implants supporting fixed prosthesis in an atrophic maxilla: A 3D-FEA biomechanical evaluation. Clin. Implant Dent. Relat. Res..

[34] Ramos A., Duarte R.J., Mesnard M. (2015). Prediction at long-term condyle screw fixation of temporomandibular joint implant: A numerical study. J. Cranio-Maxillofac. Surg..

[35] Hussein M.O., Rabie M.E. (2015). Three-Dimensional Nonlinear Contact Finite Element Analysis of Mandibular All-on-4 Design. J. Oral Implantol..

[36] Kim K.S., Kim Y.L., Bae J.M., Cho H.W. (2011). Biomechanical comparison of axial and tilted implants for mandibular full-arch fixed prostheses. Int J. Oral Maxillofac. Implants.

[37] Yazdanpanah Z., Sharma N.K., Raquin A., Cooper D.M.L., Chen X., Johnston J.D. (2023). Printing tissue-engineered scaffolds made of polycaprolactone and nano-hydroxyapatite with mechanical properties appropriate for trabecular bone substitutes. Biomed. Eng. Online.

[38] Ahmed I., Cronin P.S., Neel E.A., Parsons A.J., Knowles J.C., Rudd C.D. (2009). Retention of mechanical properties and cytocompatibility of a phosphate-based glass fiber/polylactic acid composite. J. Biomed. Mater. Res. B Appl. Biomater..

[39] Geng J.P.A., Tan K.B.C., Liu G.R. (2001). Application of finite element analysis in implant dentistry: A review of the literature. J. Prosthet. Dent..

[40] Fanuscu M.I., Vu H.V., Poncelet B. (2004). Implant biomechanics in grafted sinus: A finite element analysis. J. Oral Implant.

[41] Koca O.L., Eskitascioglu G., Usumez A. (2005). Three-dimensional finite-element analysis of functional stresses in different bone locations produced by implants placed in the maxillary posterior region of the sinus floor. J. Prosthet. Dent..

